# Global 1-km present and future hourly anthropogenic heat flux

**DOI:** 10.1038/s41597-021-00850-w

**Published:** 2021-02-22

**Authors:** Alvin Christopher Galang Varquez, Shota Kiyomoto, Do Ngoc Khanh, Manabu Kanda

**Affiliations:** Department of Transdisciplinary Science and Engineering, Tokyo Institute of Technology, Tokyo, Japan

**Keywords:** Climate and Earth system modelling, Environmental impact

## Abstract

Numerical weather prediction models are progressively used to downscale future climate in cities at increasing spatial resolutions. Boundary conditions representing rapidly growing urban areas are imperative to more plausible future predictions. In this work, 1-km global anthropogenic heat emission (AHE) datasets of the present and future are constructed. To improve present AHE maps, 30 arc-second VIIRS satellite imagery outputs such as nighttime lights and night-fires were incorporated along with the LandScan^TM^ population dataset. A futuristic scenario of AHE was also developed while considering pathways of radiative forcing (i.e. representative concentration pathways), pathways of social conditions (i.e. shared socio-economic pathways), a 1-km future urbanization probability map, and a model to estimate changes in population distribution. The new dataset highlights two distinct features; (1) a more spatially-heterogeneous representation of AHE is captured compared with other recent datasets, and (2) consideration of future urban sprawls and climate change in futuristic AHE maps. Significant increases in projected AHE for multiple cities under a worst-case scenario strengthen the need for further assessment of futuristic AHE.

## Abstract (summary)

**Table:** 

Measurement(s)	anthropogenic heat emission • anthropogenic environmental process
Technology Type(s)	satellite imaging of a planet • computational modeling technique
Factor Type(s)	global population density map • global nighttime lights map • global gridded monthly air temperature • global combustion sources map
Sample Characteristic - Environment	city
Sample Characteristic - Location	global

Machine-accessible metadata file describing the reported data: 10.6084/m9.figshare.13647935

## Background & Summary

68% of the global population is predicted to be urban-dwellers by the year 2050^1^. The rate of urbanization, commonly estimated in terms of urban population growth, differs by region with most developing nations increasing faster than developed ones. Rapid urban population increase elicits an increase in vulnerability or exposure to multiple environmental risks. These risks (e.g. heat stresses, epidemics, natural disasters) may be exacerbated by multiple factors such as air pollution, urban-induced modifications to weather, and the irreversible impacts of global climate change. Among the factors contributing to the aforesaid risks is anthropogenic heating (or anthropogenic heat emission, AHE) which is human-induced heat emitted into the atmosphere. The environmental risks that arise from AHE intensification vary per city and climate condition. Thus, spatiotemporal AHE information are essential to drive studies^2–5^ which utilize climate models to deeply understand the interaction of cities (e.g. urban heat islands) and background climate change.

To date, global or regional AHE datasets are continually being developed. Known databases include works of Flanner *et al*.^2^, Sailor *et al*.^6^, Allen *et al*.^7^ (commonly called Large scale Urban Consumption of energy or LUCY), and more recent databases of Yang *et al*.^8^, Jin *et al*.^9^, and Dong *et al*.^10^. This work aims to fill prevailing gaps in estimation and model usage of AHE, namely:

1. AHE datasets remain coarsely represented despite the improvement in spatial grid spacing (see technical validation for inter-comparison of existing high-spatial-resolution datasets);
2. Futuristic AHE maps that are in line with a city or country’s plausible response to climate change (e.g. shared-socioeconomic and representative concentration pathways) are also lacking and are limited to annual or monthly values;
3. Developing cities, remote industrial locations, or non-target cities remain underrepresented;
4. As a result of the above, spatial details of AHE and their projections are still limited in climate models.

While using the methodology of Dong *et al*.^10^ as a baseline, we constructed a present-day AHE and a future AHE datasets (collectively referred to as AH4GUC) that consider spatial changes due to projected urban sprawling, and climate change scenarios. AH4GUC (units: Watts/m^2^ or W/m^2^) is provided in monthly-typical hourly values for the present (2010s) and future (2050 s). The spatial resolution is 30 arc-seconds (~1-km). AH4GUC is an expansion and improvement of the dataset by Dong *et al*.^10^.

Relevant, robust, and freely accessible datasets were used as inputs. These datasets correspond to country-level energy consumption data, digital maps, satellite images, urban growing models, and climate model outputs which are operationally maintained to improve its accuracy and performance. Population maps such as LandScan^TM ^^11^ contain spatial information of population with 30 arc-second resolutions and is freely available for non-commercial use. The Visible Infrared Imaging Radiometer Suite (VIIRS)^12^, a scanning radiometer installed by NASA, can provide improved nighttime light distribution and improved fire detections compared to its predecessors. In this study, VIIRS nighttime lights and night-fire distributions were incorporated into the model to improve the top-down method of estimating present AHE. Two types of climate-change model outputs prescribed by standard plausible climate and socio-economic pathways (Intergovernmental Panel on Climate Change fifth assessment^13^) were used to specify future AHE scenario. The first represents radiative forcing or so-called “representative concentration pathways” (RCP)^14^. The second (commonly called socio-economic pathways or SSP^15^) defines future socio-economic conditions. A pair of RCP and SSP (based on scenario matrix^16^ concept) may be used to define future energy consumption or future monthly air temperatures. Combining a scenario with the datasets mentioned earlier, a global urban sprawl dataset, and a population growth model, future AHE (2050 s scenario defined by RCP8.5 and SSP3) map was constructed.

In summary, the highlights of the dataset are as follows:

1. Improved spatial heterogeneity of AHE distribution in urban centers with the incorporation of the VIIRS nighttime lights distribution maps of NOAA.
2. Consideration of future changes in population distribution and energy consumption through a combination of available datasets: an urban-growth probability global map; SSP model outputs of country-level population, gross domestic product (GDP), and energy consumption; and RCP model outputs of near-surface air temperatures.
3. Detection and incorporation of heat-emission point sources using open-source satellite products.

AH4GUC was verified using AHE datasets constructed using the bottom-up approach, and recent present and futuristic AHE datasets. Results reveal improved representation in downtown commercial areas showcasing more spatial heterogeneity, unlike other existing datasets. Confirmed using satellite images (e.g. ESRI and Google), AHEs in remote regions were also captured in detail in the AH4GUC unlike other existing datasets. Unlike other future datasets, the dataset considers horizontal urban sprawl (i.e. population growths at previously non-urbanized locations). In the future, developed cities, tending to have higher AHE, will emit lower anthropogenic heat in the 2050 s. On the other hand, AHE will increase for low-income but rapidly developing cities. Overall, the global increase in AHE remains to be expected.

AH4GUC may be used to feed global climate models increasingly capable of running finer spatial resolutions. More importantly, the dataset will aide in the developmental planning of cities offering first-hand information of high AHE locations for any city or town of interest. The simple model presented herein can also be used to construct other future AHE scenarios.

## Methods

AH4GUC was created to meet three objectives. First, improvement and construction of a present-state high-spatial-resolution AHE dataset developed by Dong *et al*.^10^. The second objective is the development and application of a methodology for estimating future distribution (2050 s) of AHE. Third is to incorporate a dataset that enables the detection of point sources of AHE.

### General framework

**Fig. 1:**
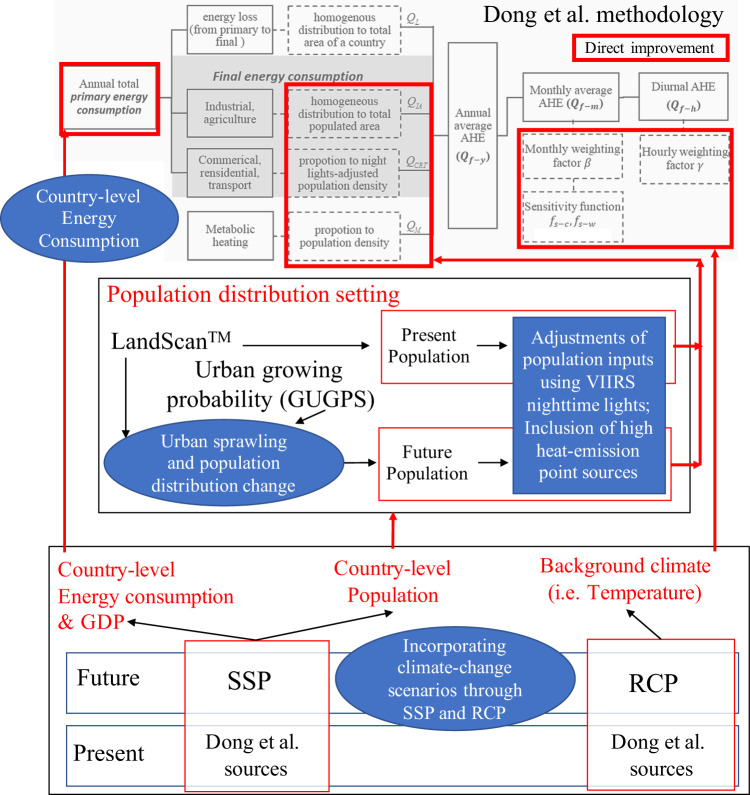
Workflow of AH4GUC construction. AH4GUC directly influences the components bounded by red boxes in the methodology of Dong *et al*.^10^, region in grey (figure used with permission).

AHE datasets are constructed either through a “top-down” approach or a “bottom-up” approach. The main difference between them is in the scale of known input information. For the “top-down” approach, usually, energy consumption is coming from a regional/country-level and then scaled down into gridded information. For the “bottom-up” approach, it either utilizes actual AHE measurements or known energy consumption information for specific sites, scaled-up to its gridded value. Although more accurate, AHE using “bottom-up” approaches are difficult to implement and update due to the lack of actual measurements or energy consumption information for points within grids. In this study, we follow the “top-down” approach of Dong *et al*.^10^ (Top of Fig. 1 inside grey-filled region). As with other top-down approaches^2,7^, the law of energy conservation is assumed in the methodology, such that all energy produced is eventually released to the atmosphere within the global domain. The energy consumed is later emitted into the atmosphere as heat, and this process occurs almost simultaneously. Under this assumption, annual-average anthropogenic heat of the country (*Q*_*f−y*_) is assumed to be equal to the sum of the primary energy consumption and anthropogenic heat from metabolic processes (*Q*_*M*_). Primary energy consumption of the country can be further partitioned to components such as “heat loss” (*Q*_*L*_), and AHE of “industrial and agricultural” (*Q*_*IA*_), and “commercial, residential, and transport sectors” (*Q*_*M*_) (Eq. 1).

1 $${Q}_{f-y}={Q}_{L}+{Q}_{CRT}+{Q}_{IA}+{Q}_{M}$$

The country-level (bulk) quantities of the components in Eq. 1 can be obtained by using energy balance statistics that relate the amount of consumption per component to the total primary energy consumption. Energy balance statistics provided by the International Energy Agency (IEA) were used (see Table 1). Various settings to spatially allocate the AHE from each sector were defined in Dong *et al*.^10^.

**Table 1:** Model Inputs.

Input Datasets	Name/Description	Resolution
Population	LandScan^TM ^^36^	30 arc-second (appx. 1-km)
Nighttime Lights	VIIRS DNB Nighttime Lights^37^	30 arc-second (appx. 1-km) resampled to LandScan^TM^
Nightfire	VIIRS Nightfire verson 3.0 (Gravite)^38^	30 arc-second (appx. 1-km) resampled to LandScan^TM^
Energy Consumption and Energy Intensity	Energy Balance Statistics from the International Energy Agency (IEA)^39^	Country-level
	Shared-socioeconomic Pathways (SSP) by The International Institute of Applied Systems Analysis (IIASA)^40^	Regional-level
	Energy intensity level of primary energy (MJ/$2011 PPP GDP)^33^	Country-level
Monthly Surface Air Temperature	NCEP/NCAR 40-Year Reanalysis Project (2012 output)^41^	2.5 arc-degrees
	Climate, Environmental Retrieval, and Archive (CERA); The World Data Center for Climate (WDCC)^32^	1~2.5 arc-degrees, varying (Supplementary Table S1)

The workflow of this study is shown in Fig. 1. All inputs used in this study are listed in Table 1. The improvements to the existing AHE dataset^10^ and the method to construct the futuristic AHE are contained outside of the grey-filled region (i.e. methodology of Dong *et al*.^10^) of Fig. 1. The following sub-sections (blue-filled region) represent the specific processes to update the work of Dong *et al*.^10^. The sub-sections, “Adjustments of population inputs using VIIRS nighttime lights” and “Inclusion of high heat-emission point sources”, discuss how the present and future population input estimates the *Q*_*CRT*_, *Q*_*IA*_, and *Q*_*M*_ in AH4GUC. The bulk country-level values of population, energy consumption & GDP, and the background climate that affects the future population distribution setting, future country-level primary energy consumption, and future temporal weighting factors of AHE, respectively, are discussed in the sub-section, “Incorporating climate-change scenarios through SSP and RCP”. The procedure to estimate the future distribution of population is discussed in the sub-section “Urban sprawling and population distribution change”. The sub-section “Country-level energy consumption” is also discussed to mention how country-level primary energy consumption can be estimated from the SSP. Each sub-section is also situated under the time period of the AHE construction it applies.

### Construction of present scenario

#### Adjustments of population inputs using VIIRS nighttime lights

LandScan^TM^ (2013)^11^ and Global Radiance Calibrated Products of the Defense Meteorology Satellite Program/Operational Linescan System (DMSP-OLS)^17^ were previously used by Dong *et al*.^10^ to represent the population and nighttime lights at 1-km spatial resolution, respectively. The Global Radiance Calibrated Products of DMSP-OLS are no longer updated and was replaced with the monthly-available composite data from the Visible Infrared Imaging Radiometer Suite carried by the Suomi National Polar-orbiting Partnership (VIIRS) nighttime lights^18^, a more accurate dataset^19,20^ than then DMSP-OLS nighttime lights.

Nighttime lights intensity is needed to adjust the population density dataset. The LandScan^TM^ dataset was found to mainly indicate population of residential areas. Using this raw population alone may underestimate final AHE of commercial areas. Nighttime lights, which are of higher intensity at commercial areas, were used to adjust the population map to assign new weighting parameters when downscaling country-level AHE into each grid.

Average radiance composite images (v1) from the VIIRS Day/Night Band from the period January 2014 to March 2016 (period available during the time the research was conducted) were downloaded and resampled using spatial averaging to the resolution of LandScan^TM^. Unlike the Global Radiance Calibrated Products of DMSP-OLS (nanowatts/cm^2^/sr), the range of values (watts/cm^2^) vary from one image to another. A single input nighttime-lights dataset (henceforth referred to as nVIIRS) was acquired by calculating the 20% trimmed mean of all images for each grid (or pixel) to capture the general nighttime light condition.

The derived nighttime-lights dataset was used to adjust the population density information calculated from LandScan^TM^. The steps are as follows:

1. Estimate a linear regression curve between both datasets within the same country boundary.
2. Using the Tukey’s method, detect outliers and calculate a second linear regression from the non-outliers.
3. Population value at the outlier grids having nighttime light intensity above a certain threshold are replaced with a new population value calculated using the second linear regression.
4. Repeat the process for all grids and countries.

In the above procedure, the threshold nighttime light value was set to vary per country in order to consider intercountry differences. Because of the units and dynamic range of the nVIIRS, the lower threshold value (600) for the population-adjustment algorithm used in Dong *et al*.^10^ is not applicable. After inspecting multiple downtown commercial areas of capital cities, it was also found that setting an absolute threshold value may limit downtown detection for countries that have generally lower nighttime lights detection (e.g. Jakarta or Manila). To ensure intercountry variability, percentile thresholding using all grids’ nighttime light samples within a country were used to determine a country-specific threshold. To prevent over adjustment outside of commercialized cities, a percentile threshold value of 99.9% was used. The whole procedure results to a new global distribution of population-adjusted by nighttime light values that exhibit higher in commercial areas.

#### Energy consumption and socio-economic inputs

In the work of Dong *et al*.^10^, the annual total primary energy consumption of each country was taken from the U.S. Energy Information Administration (EIA) (see Table 1). In this study, this information was taken from the SSP database (The International Institute of Applied Systems Analysis, IIASA). The main purpose for using the energy consumption information of the SSP database is to provide consistency between energy consumption used as input for the present and future AHE estimates.

Acquiring the energy consumption values from the SSP database for each country was not straightforward because of certain limitations which include: (1) various providers providing different outputs for the same SSP scenario exists and (2) the SSP database for total energy consumption are only available regionally (i.e. group of neighboring countries). To address the variability of SSPs from various providers, an ensemble average of multiple model outputs (AIM, GCAM4, IMAGE, and MESSAGE) was calculated for the same level of SSP. Lack of country-based information was addressed using the concept of “energy intensity” which relates gross domestic product (GDP), a country-based parameter available in the SSP database (see section on *Incorporating climate chance scenarios through SPP and RCP*), with energy consumption. Energy intensity (*m*) has the units of energy consumption (TJ) per unit of GDP (purchasing power parity, billion US$2010/year). High values of *m* mean relatively high industrial energy output in proportion with GDP. In this study, we used the definition of “energy intensity” to estimate energy consumption of each country by multiplying GDP with a country’s *m*. Country-level *m* information was taken from The World Bank for the year 2010. Country-level total primary energy consumption was downscaled from regional-levels values using the following equation.

2 $${EC}_{p,c}^{yr,SSP}=E{C}_{p,r}^{yr,SSP}\times \left(\frac{{m}_{c}^{2010}{{\rm{GDP}}}_{c}^{yr}}{{\sum }_{x=1}^{{n}_{c\in r}}\,{m}_{x}^{2010}{{\rm{GDP}}}_{x}^{yr}}\right)$$

where *EC*_*p*_ corresponds to the primary energy consumption; and the indices *yr*, *c*, *r*, *SSP* corresponds to year, country, region, and SSP level, respectively. The assumption of using *m* of 2010 was based on an earlier analysis revealing negligible changes in the energy intensity from 2010 to 2015 (latest available report from World Bank).

### Construction of future scenario

#### Urban sprawling and population distribution change

To estimate a 1-km future distribution of AHE, a 1-km spatial projection of population density for a specified period is needed. Its projections must also be consistent with the known total projections for each country (available from the SSP database). In this study, distributed population projection was achieved using two steps:

1. extract 1-km urbanization probability for the future from a global urban growing projection dataset;
2. construct a model to downscale the country-level population projection into 1-km grids using the projected urbanization probability and present population density distribution. The grid positions match the AHE for the present condition.

Spatiotemporal changes in population density coincides with urban growing, which occurs either horizontally (e.g. conversion of vegetated land to an urban structure) or vertically (e.g. demolishing smaller buildings in place of taller ones). Information on futuristic urban growth can be used to project population. During the construction of AH4GUC, a high-spatial-resolution global urbanization probability map^21^ (GUGPS) was publicly available and was then used in the work. The resolution coincides with that of the population dataset used in this study and that of Dong *et al*.^10^. This conveniently removes the extra work of resampling either dataset. A specific feature is that the mapped urban growing projections are in terms of integer-type probability values (i.e. not simply urban or non-urban) for a non-urban grid to become urban for a year in the future. GUGPS was created using an urban growing model called SLEUTH^22^, an urban growing model that utilises growth coefficients finely calibrated in the model from known geospatial information (i.e. topographic slope, land cover, restricted areas for urbanization, urban cover, transportation networks, and hill-shade for visualization) of a target region. In the GUGPS implementation of SLEUTH, the target regions were represented by tiles containing cities (i.e. urban growth rates are unique to each tile).

High urbanization probability values for a location in GUGPS map means a higher chance for the said location to urbanize. A higher chance for urbanization also translates to a higher population increase under the trivial assumption that with urbanization comes population increase. We estimated future spatial projection of population density by combining the annual urbanization probabilities from GUGPS with the most recent population density data of LandScan^TM^. Furthermore, country-level population from the SSP was used as the constraint. The method^23^ is further explained.

To predict the distribution of future population density, a discretized form of the logistic model^24^ was implemented for each grid of the same country as follows,

3 $${P}_{i,j}^{n+1}=\frac{\left({A}_{i,j}^{n}+1\right){P}_{i,j}^{n}}{1+{B}_{i,j}^{n}}$$

4 $${A}_{i,j}^{n}=\exp \left({r}_{i,j}^{n}\right)-1$$

5 $${B}_{i,j}^{n}=\frac{{A}_{i,j}^{n}}{{K}^{n}}$$

where *P* refers to the population value at the coordinates *n*, *i*, and *j*, referring to the year (discrete), longitudinal, and latitudinal coordinates, respectively. Parameters *r* and *K* are the natural population growth and the limiting values for population growth for each grid, respectively. They are assumed, in this study, to inherently represent population influx, net increase (births and deaths) of people, and inter-decadal factors (change in infrastructure or policies) influencing population growth. *K* was set to the maximum population density value of all grids within the same country for the year 2013. On the other hand, *r* was assumed to be a linear function of the urbanization probability ${f}_{i,j}^{n}$ outputted from SLEUTH using the following equations,

6 $${r}_{i,j}^{n}=\left\{\begin{array}{l}{a}^{n}\;{f}_{i,j}^{n+1}\quad 0 < {P}_{i,j}^{n} < 100\\ {b}^{n}\;{f}_{i,j}^{n+1}\quad \;\;100\le {P}_{i,j}^{n}\end{array}\right.$$

where *a* and *b* are grid-varying internal parameters for future years obtained by minimizing the root-square difference of the country-level total estimated ${P}_{i,j}^{n}$ for *n* = 2001 (starting period), 2006, 2009, 2010, 2012 and their corresponding real values taken from LandScan^TM^. An additional constraint for the minimization was also set such that the absolute percentage error between the country-level total of estimated ${P}_{i,j}^{n}$ and those from the SSP should not exceed 1%. *a* and *b* were then recalculated for every decadal interval (i.e. *a* and *b* were set to the same value from the period 2041 to 2050). Also, grids $\left({P}_{i,j}^{n}=0\right)$ that will be affected by urban encroachment $\left({f}_{i,j}^{n+1}=0\right)$ was initialized with a ${P}_{i,j}^{n+1}$ value of 10.

Future prediction of nighttime lights was not conducted for two reasons. First, it is uncertain how country-level indicators from SSP and urban sprawl quantitatively relate with nighttime lights intensity. Second, nighttime lights were mainly used to adjust the population distribution to have more representation in commercial areas. Commercial areas are assumed to not change up to the target future period. Lastly, urban shrinking was also not modeled in this study since depopulation does not necessarily result in removal of urban cover.

#### Incorporating climate-change scenarios through SSP and RCP

As briefly described earlier, Integrated Assessment Models (IAM) and global climate models (Coupled Model Intercomparison Project Phase 5 or CMIP5), which utilizes SSP and RCP as inputs, model the socio-demographic and climate information that are needed to estimate the future AHE. From SSP, projections of country-level energy consumption at decadal intervals can be obtained using the method introduced earlier (see section on *Energy consumption and socio-economic inputs*). In addition, projections of country-level total population at decadal intervals are also available and were used to estimate the future spatial distribution of population for each country (see section on *Urban sprawling and population distribution change*). Meanwhile, monthly mean near-surface air temperature (*tas*) projections were also downloaded from the CMIP5 models. The monthly mean values of *tas* are used to downscale the annual-average AHE to its monthly values.

In this study, we utilized the SSP3, which assumes a region experiencing high mitigation and adaptation challenges to climate change. From the SSP, country-based population and GDP projections are available. The population was taken from the SSP3 modeled by the International Institute for Applied Systems Analysis (IIASA), the National Center for Atmospheric Research (NCAR). GDP was taken from the ensemble average of GDP projections of SSP3 modeled by The Organization for Economic Cooperation and Development (OECD: 177 countries estimated) and IIASA (150 countries estimated).

*tas* from the RCP8.5 (high climate forcing) was taken from the ensemble average of all model outputs maintained by the Climate, Environmental Retrieval, and Archive (CERA) in The World Data Center for Climate (WDCC). The RCP8.5 models used are listed in Supplementary Table S1.

In the future AHE dataset, energy-consumption data were missing for a few countries and districts. Thus, future AHE distribution were unavailable for North Korea, Taiwan, Guam, South Sudan, Seychelles, Western Sahara, São Tomé and Príncipe, French Guiana, Carribean islands east of Puerto Rico, Falkland Islands, and a few islands including and surrounding American Samoa.

Scenarios were finally constructed from the combination of SSP and RCP types. Using the concept of climate scenario matrix^15^, a “worst-case” scenario was constructed under RCP8.0-SSP3. This scenario assumes that the future AHE is an integrated result of minimal global efforts to adapt and mitigate climate change.

### Inclusion of high heat-emission point sources

The top-down approach, which mainly utilizes bulk energy consumption quantities and population density distributions, tends to overestimate the AHE compared with the bottom-up approach. One of the possible reasons is that few districts exist which emit significant amounts of anthropogenic heat without necessarily having very large population density values or extremely high nighttime light intensity. These locations include manufacturing buildings and power plants. In this study, a simplified approach of including the detected combustion sources in the AHE estimation is introduced. These potentially high AHE districts were detected and assigned additional weighting to the grid allocation of country-level AHE. Detection of high-heat emission point sources were conducted using the VIIRS night fire datasets (VIIRS-Nightfire)^25^ (see Table 1).

VIIRS-Nightfire is a product of VIIRS which contains information regarding the day-to-day location of detected combustion sources. For example, flared gas volume is detected in VIIRS-Nightfire which are usually attributed to refineries and liquified natural gas terminals^26^. Version 3.0 (GRAVITE) of the VIIRS-Nightfire, the latest available version when the study was conducted, was downloaded for the period December 2017 until February 2018 from the database (Table 1). The following filtering procedure was necessary to detect the close-to-permanent combustion sources (flared gas volume) in the AHE estimation:

1. Using the geographical location (latitude and longitude) of the combustion sources, daily occurrences of combustion within a 30-arc second grid are summed and stored as the grid’s scalar value.
2. Temporary heat sources detected by VIIRS are removed by setting a threshold value of the grid’s frequency of combustion occurrence to 3. Grid’s with less than or equal to 3 days of combustion occurrence (e.g. biomass burning, moving heat sources, fire incidents) were assumed to be temporary or artificial, and not considered in the AHE estimation.

After conducting the above procedure, a global distribution of daily frequencies of combustion sources ${d}_{i,j}^{hot}$ was obtained. ${d}_{i,j}^{hot}$ was assumed as a weighting for the amount of anthropogenic heat in the detected combustion source relative to the other sources within the same country. The same ${d}_{i,j}^{hot}$ was used to estimate the future AHE distribution. This means that existing refineries or liquified natural gas terminals of a country were assumed to operate in the same manner until 2050. Equation 1 was modified and the grid value for each AHE component is now estimated as follows,

7 $${Q}_{f-y}={Q}_{L}+{Q}_{CRT}+{Q}_{IA}+{Q}_{M}+{Q}_{point}$$

8 $${\left({Q}_{point}\right)}_{i,j}=\left\{\frac{0.1\times \left({R}_{CRT}+{R}_{IA}\right)\times {d}_{i,j}}{\sum {A}_{i,j}\times \sum {d}_{i,j}}\right\}\times \frac{E{C}_{p}}{T}$$

9 $${\left({Q}_{L}\right)}_{i,j}=\left\{\frac{{R}_{L}-0.1\times s\times \left({R}_{CRT}+{R}_{IA}\right)}{\sum {A}_{i,j}}\right\}\times \frac{E{C}_{p}}{T}$$

10 $${\left({Q}_{CRT}\right)}_{i,j}=\left\{\frac{{R}_{CRT}\times P{D}_{i,j}^{\ast }}{{A}_{i,j}\times \sum P{D}_{i,j}^{\ast }}\right\}\times \frac{E{C}_{p}}{T}$$

11 $${\left({Q}_{IA}\right)}_{i,j}=\left\{\frac{\left[{R}_{IA-}0.1\times \left(1-s\right)\times \left({R}_{CRT}+{R}_{IA}\right)\right]\times {A}_{i,j}^{{\prime} }}{{A}_{i,j}\times \sum {A}_{i,j}^{{\prime} }}\right\}\times \frac{{EC}_{p}}{T}$$

Each component is estimated per grid of position (*i, j*) and are based on the ratio of its final energy consumption to the total primary energy consumption (*EC*_*p*_) calculated from the energy balance statistics (EIA database). *R*_*L*_, *R*_*CRT*_, *R*_*IA*_ are the respective ratios of the final energy consumption (allocated to “heat loss”, “industrial and agricultural”, and “commercial, residential, and transportation” sectors) to the total primary energy consumption within each country. *A*_*i,j*_ refers to the area (m^2^) of the grid. *A*′_*i,j*_ corresponds to the area of a populated grid. When population density is greater than 0, *A*′_*i,j*_ is set to *A*_*i,j*_. On the other hand, *A*′_*i,j*_ was set to 0 for uninhabited grids. PD^*^_i,j_ is the value of the population density adjusted by nighttime lights (see section on *Population and nighttime lights consideration*) at grid (*i*, *j*).

Equation 8 was based on previous findings^27,28^ that waste heat from point sources such as power stations and incineration plants account for 10~11% of the final energy consumption. Here, we set the allocation for *Q*_*point*_ to be 10% of a country’s final energy consumption. To conserve the total primary energy consumption with the introduction of *Q*_*point*_, adjustments were made to *Q*_*L*_ and *Q*_*IA*_. The adjustments were made based on the energy consumption by industrial sectors and the energy losses (i.e. waste heat). *s* (Eqs. 9 and 11) is the ratio between the [amount of final energy consumption allocated to “heat loss”] to the [sum of this value and the final energy consumption allocated to “industrial sectors”]. *s* determines the adjusted values for *Q*_*L*_ and *Q*_*IA*_ in order to compensate for the inclusion of *Q*_*point*_. *EC*_*p*_ is in the units of Joules (J) and *T* corresponds to a 1-year period in terms of seconds (s).

## Data Records

AH4GUC is a database which contains maps of hourly-representative anthropogenic heat emissions (AHE) representing the periods 2010s and 2050 s. The AHE of 2050 s represents a single future scenario based on RCP8.5 and SSP3 climate change pathways. This future “worst-case” scenario assumes minimal or no adaptation and mitigation measures implemented in all countries. Monthly-averaged and annual-averaged values representing the 2010s and 2050 s are also provided. All files are available in single-band raster format (GeoTIFF format) are freely accessible through Figshare^29^ and at a university-maintained server (Tokyo Institute of Technology, http://urbanclimate.tse.ens.titech.ac.jp/). Each pixel contains integer formats of AHE in units of W per sq. m. per 100,000. GeoTIFFs can be further analyzed or process using any GIS software or programming tools, such as R or Python.

## Technical Validation

AH4GUC was verified using multiple existing AHE datasets created using either “top-down” and “bottom-up” approaches (see *Methods*). The AHE map of the 2010s was compared with the “bottom-up” datasets of Moriwaki *et al*.^30^ and Iamarino *et al*.^31^ prepared for Tokyo and London, respectively. The dataset was also compared with other recently released “top-down” datasets, such as the AH-DMSP^8^, PF-AHF^9^, and the dataset of Dong *et al*.^10^ (DONG). For the 2050 s scenarios, future scenarios from PF-AHF and Flanner^2^ (FL) were used for its validation. Focusing mainly on the intensity and spatial distribution, the annual-averaged AHE values were mainly used. Since no modifications in the methodology^10^ were implemented to the estimation of monthly and hourly values, readers are advised to refer to Dong *et al*.^10^ for its verification. In this section, we verify AH4GUC in terms of its performance relative to other datasets when inspecting multiple large cities across varying spatial resolutions and zonal/meridional means.

**Fig. 2:**
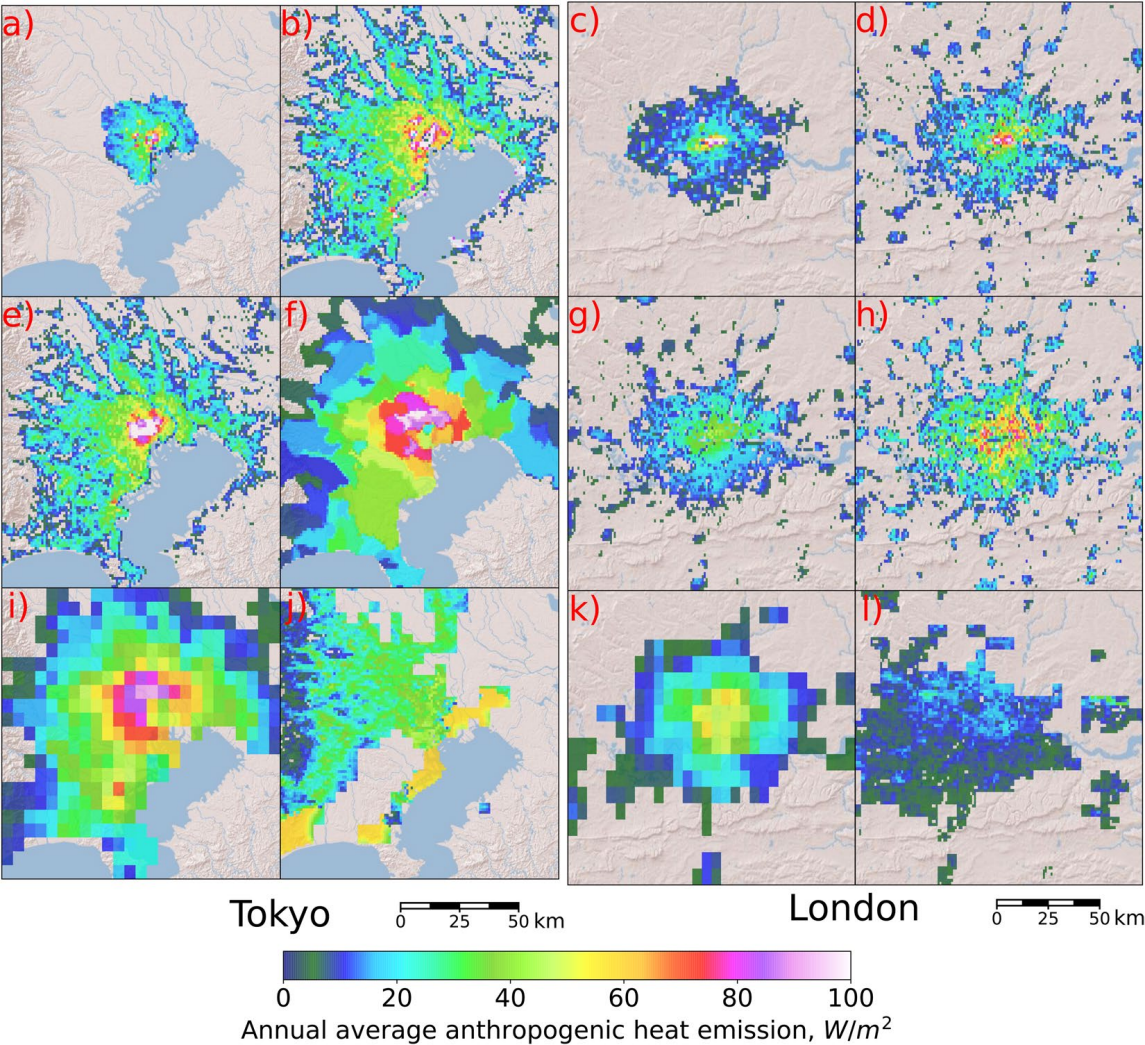
Comparison of anthropogenic heat emission datasets for Tokyo and London. “Bottom-up” approaches by (**a**) Moriwaki *et al*.^21^ and (**c**) Iamarino *et al*.^22^ for Tokyo and London, respectively; (**b,d**) AH4GUC; (e,g) DONG^10^; (**f,h**) PF-AHF^9^; (**i,k**) FL^2^; (**j,l**) AH-DMSP^8^. Values lower than 5 W/m^2^ are masked.

The most important comparison is whether the datasets constructed from “top-down” approaches (i.e. “top-down” datasets) compare reasonably with the “bottom-up” approaches (i.e. “bottom-up” datasets). Present-day (2010s) annual-averaged AHE from various datasets were mapped in their finest available native resolutions for Tokyo and London as shown in Fig. 2. Both cities were selected for the availability of “bottom-up” datasets. The Tokyo^21^ and London^22^ “bottom-up” datasets (Fig. 1a,c) correspond to AHE of 1997 and 2008, respectively. Since the “bottom-up” datasets were constructed from actual heat sources (i.e. records of actual building locations and individual energy consumption, traffic), we assumed that they represent a more accurate spatial distribution than those from the “top-down” approaches but are difficult to update given the stringent requirement of actual heat sources and intensities. The “top-down” datasets, except for AH-DMSP, mostly capture the relatively higher emissions at the city-centers. Both AH4GUC and DONG capture spatial heterogeneity of AHE quite well in Tokyo, while AH4GUC, DONG, and PF-AHF represent better London’s AHE.

In Tokyo (Fig. 2a,b,e,f,i,j), the AHE values at the city center (reddish and white region at the center of Fig. 2a) appear slightly more dispersed in AH4GUC (Fig. 2b) than DONG (Fig. 2e). Higher spatial heterogeneity can be found, especially at the city-center, for AH4GUC better resembling the “bottom-up” dataset, whereas DONG appears to have higher values spread uniformly at the city center. The city center of Tokyo seats the Imperial Palace which is largely surrounded by parks and vegetated land (i.e. low AHE). The main reason for their differences can be attributed to the replacement of DMSP-OLS with the VIIRS nighttime lights dataset in the construction of AH4GUC. For the same city, the highest available resolutions of PF-AHF (Fig. 2f) and FL (Fig. 2i) were not enough to capture spatial heterogeneity of the city-center. The main reason for this was that the population map used to downscale the energy consumption data into AHE of each grid was not sufficient to capture the AHE, especially, in the commercial areas. PF-AHF and FL appear to slightly shift the high AHE region northwest and outwards from the city center. This region represents a relatively high residential population, which means globally-available population datasets tend to reasonably capture the residential population more than the locations of the populace during the daytime (i.e. workplaces, offices, commercial areas). Also, despite the high-spatial resolution of the PF-AHF, its estimated AHE distribution for Tokyo appears uniformly distributed for grids within the same administrative boundaries (i.e. the same population value was assigned for grids within the same administrative boundaries). This implies the lack of detailed spatial representation of the population in the region. Overall comparing the grid values at grids with “bottom-up” information for Tokyo, the Pearson correlation coefficients for DONG, AH4GUC, AH-DMSP, FL, and PF-AHF are 0.697 (two-tailed p-value: 2.5E-139), 0.719 (4.0E-155), 0.003 (0.91), 0.003 (0.92), and 0.182 (1.E-8), respectively.

In London (Fig. 2c,d,g,h,k,l), the “top-down” datasets generally show a wider area of large AHE values than the “bottom-up” dataset (Fig. 2c). These larger estimates by the “top-down” datasets were mainly attributed to the difference in the target period of estimation. At the city-center (reddish and white region at the center of Fig. 2c), the improvement of AH4GUC (Fig. 2d) was noticeable compared with DONG (Fig. 2g) in terms of the representation of AHE values at the city-center. Similar to the comparisons in Tokyo, PF-AHF also depicted higher values at the immediate surroundings of the city-center compared to DONG but relatively lower values at the city-center were estimated. This suggests the lack of daytime representation (when people are generally situated in the workplace such as in commercial areas located at the city-center) from the global population dataset. However, unlike Tokyo, spatial heterogeneity of AHE values was captured by the PF-AHF (i.e. no uniformity of values within the same administrative boundaries). The coarse resolution of FL also failed to capture intense AHE values at the city-center revealing the same tendency as PF-AHF (see visual comparisons of “top-down” datasets resampled using averaging to the same resolution as FL). Overall comparing the grid values at grids with “bottom-up” information for London, the Pearson correlation coefficients for DONG, AH4GUC, AH-DMSP, FL, and PF-AHF are 0.667 (two-tailed p-value: 0.0), 0.78 (0.0), 0.526 (4.3E-211), 0.574 (3.4E-260), and 0.34 (3.3E-81), respectively.

**Fig. 3:**
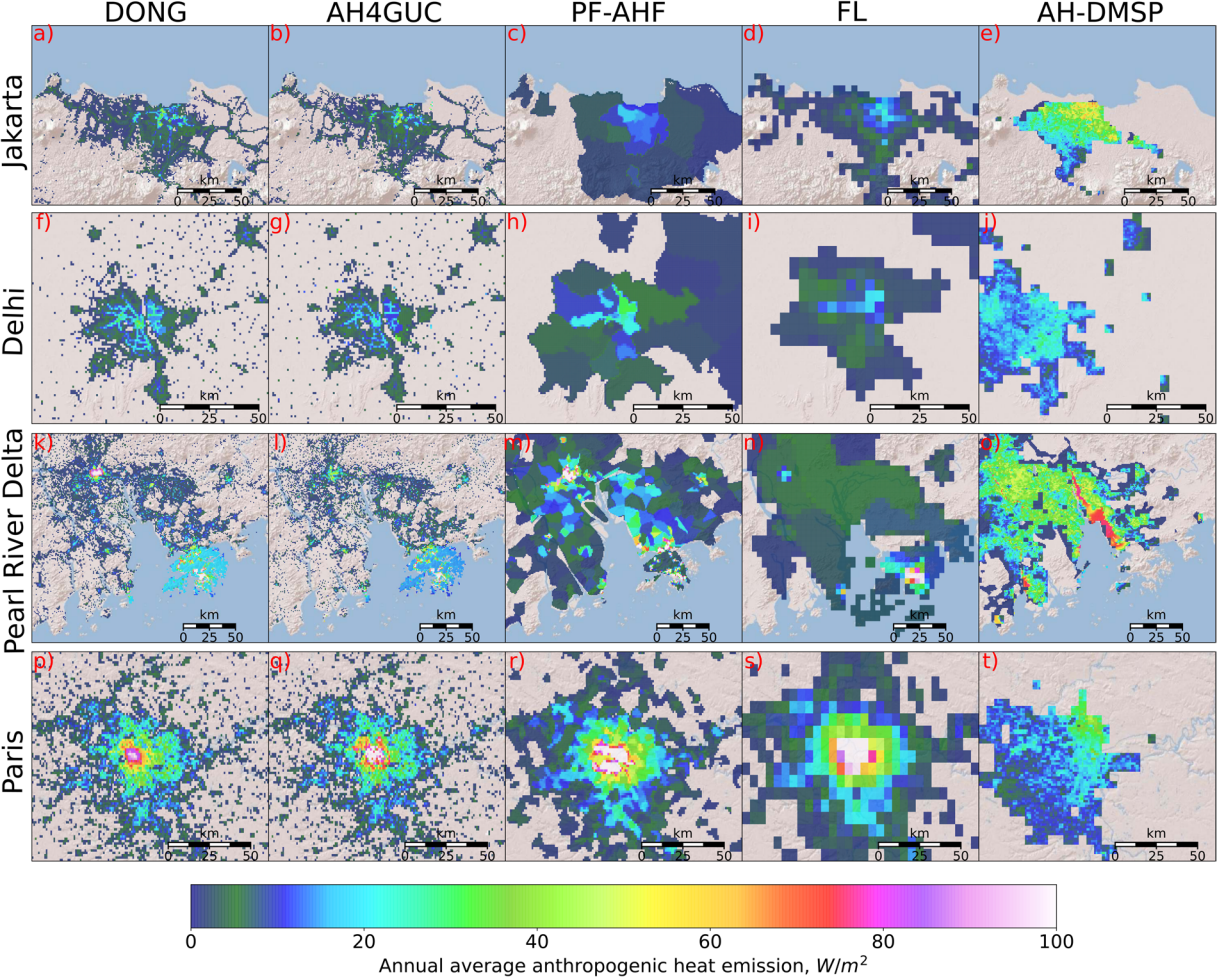
Comparison of global anthropogenic heat emission datasets for selected megacities. Figures on the same row correspond to cities: Jakarta (**a,b,c,d,e**), Delhi (**f,g,h,I,j**), Pearl River Delta (**k,l,m,n,o**), Paris (**p,q,r,s,t**). Figures on the same column correspond to the datasets: DONG^10^ (**a,f,k,n**), AH4GUC (**b,g,l,q**), PF-AHF^9^ (**c,h,m,r**), FL^2^ (**d,i,n,s**), AH-DMSP^8^ (**e,j,o,t**). Values lower than 1 W/m^2^ are masked.

During the time of this investigation, the AH-DMSP contained an inherent error where AHE appeared shifted westward throughout the whole global domain (as evident from Figs. 2 and 3). Disregarding this inherent error, AH-DMSP still showed the poorest resemblance with the “bottom-up” datasets (Fig. 2 and Fig. 3). The main reason behind this was that among the globally available “top-down” datasets, the AH-DMSP did not primarily use population maps as a weighting factor to downscaling energy consumption of the country (i.e. population was used for masking above a certain threshold). Nighttime lights information is not enough to represent AHE.

The “top-down” datasets were also compared in the same manner for other megacities as shown in Fig. 3. “top-down” datasets resampled to the same resolution as FL using averaging are shown in Supplementary Figure S1. In summary, the same tendency could be said as with the comparisons in Tokyo and London. For most cities, little differences were found in DONG and AH4GUC when viewed over wider domains. However, the differences become more apparent at the city-center (where DONG tends to be slightly more uniform) and at detected point sources (see *Methods*; also discussed later). As confirmed early in the discussion of Tokyo and London, the improvement caused by the replacement of DMSP-OLS with VIIRS is quite obvious at the city-centers. Commercial areas characterized by clusters of tall buildings have lower (higher) values than the residential areas in DONG (AH4GUC). In Jakarta for example, the intensity of lights from the DMSP-OLS were all below the fixed nighttime-lights threshold for adjustment in DONG which resulted in its underestimation of AHE in commercial areas (Supplementary Figure S2).

As with Tokyo and London, the level of spatial detail throughout the cities was not well captured by PF-AHF, FL, and AH-DMSP (Fig. 3) when compared with DONG and AH4GUC. Such that, AHE mapped from PF-AHF appears uniformly distributed at grids within the same administrative boundary despite its high spatial-resolution. Likewise, FL shows spatial heterogeneity adequate to its level of resolution, while occasionally featuring uniform distribution at grids within the same administrative boundary. AH-DMSP was found to be different from the other “top-down” datasets with their centers shifted geospatially westwards from the city-center.

**Fig. 4:**
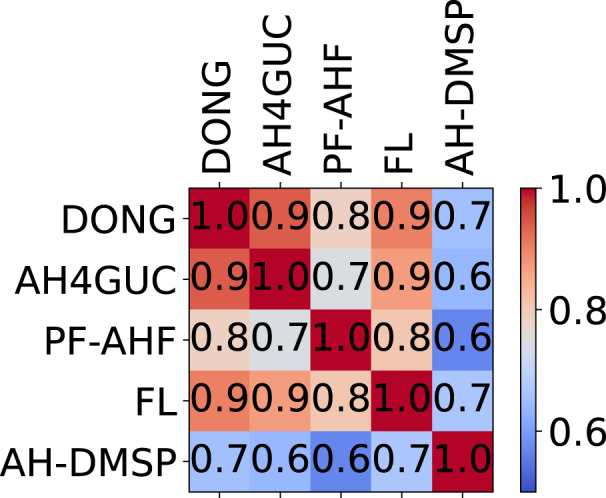
Matrix of Pearson correlation coefficients between “top-down” datasets of samples at a global scale. All datasets were resampled to the same resolution of FL using averaging.

**Fig. 5:**
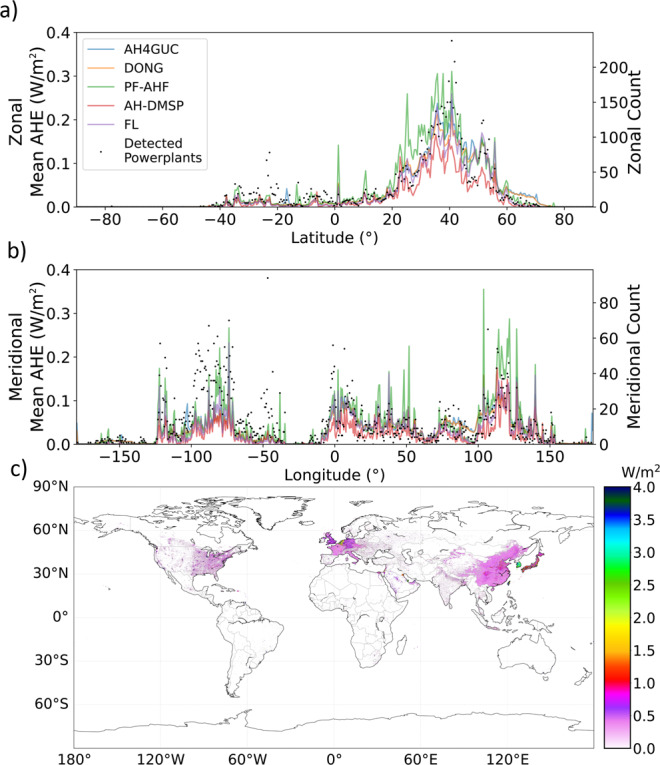
Zonal mean (**a**) and meridional mean (**b**) of anthropogenic heat emission (left-hand side vertical axis) and quantity of detected powerplants^35^ (right-hand side vertical axis). All datasets resampled to FL resolution (0.5°) and statistics estimated at 0.5° intervals. (**c**) shows a global map of AH4GUC for 2010s in native resolution.

The Pearson correlation coefficients comparing the selected “top-down” datasets with each other are summarized on Fig. 4. Generally, AH4GUC tends to spatially correlate well with other datasets. However, differences in mean AHE estimates can be seen. The global average AHE of the present period by DONG, AH4GUC, PF-AHF, FL, and AH-DMSP are 0.030 W/m^2^, 0.031 W/m^2^, 0.039 W/m^2^, 0.027 W/m^2^, and 0.018 W/m^2^, respectively. The slight global increase of AHE by 0.001 W/m^2^ in the AH4GUC compared to DONG is mainly attributed to the nighttime lights adjustment which concentrates the downscaled AHE values at urban areas especially at large administrative boundaries. This higher global average value of AH4GUC also appears in PF-AHF. The global averages can be further decomposed by mean values of AHE across the same latitude and longitude (Fig. 5). We defined zonal (meridional) mean to refer to the spatial mean of AHE across the same latitude (longitude) bins of 0.5° intervals. In this latitudinal and longitudinal analyses, all datasets were resampled via averaging to match the same spatial resolution as FL (the version with 0.5° resolution). Zonal and meridional sum of powerplants (dataset prepared by the Global Energy Observatory^23^) are also overlain on Fig. 5.

Comparing the “top-down” datasets across latitudes and longitudes reveal similarities and differences. All datasets generally suggest significantly large AHEs at regions located at the midlatitudes (20° to 60° latitude; Fig. 5a) and longitudes corresponding large continents such as North America (−50°~−120° longitude), Europe (−10°~60° longitude), and Asia (Fig. 5b). The zonal and meridional means of AHE also generally coincide with the total number of geolocated powerplants listed in the Global Powerplant Database^23^. It is to be carefully noted that not all powerplants around the world are geolocated here. Consistent with the calculated global average, AH4GUC and PF-AHF generally have higher values than the other datasets throughout the latitudinal and longitudinal averages, while PF-AHF (green lines in Fig. 5) tends to have lower estimates of AHE. From the meridional mean, it can be seen that the larger peaks alternate between AH4GUC and PF-AHF, while much larger peaks are apparent in the PF-AHF.

At the latitude range of −40°~40°, PF-AHF shows relatively larger peaks compared with the other datasets. On the other hand, AH4GUC and DONG generally have larger values at northern areas of the midlatitudes. From the meridional mean (Fig. 5b), it can be seen that these large values appear more obvious over regions in Russia, Canada and Europe. Zooming in through these regions such as Russia (shown in Supplementary Figures S3–S5), it appears that the detailed LandScan^TM^ and nighttime lights allows more detailed detection AHE at industrial locations not necessarily listed in the Global Powerplant Database.

**Fig. 6:**
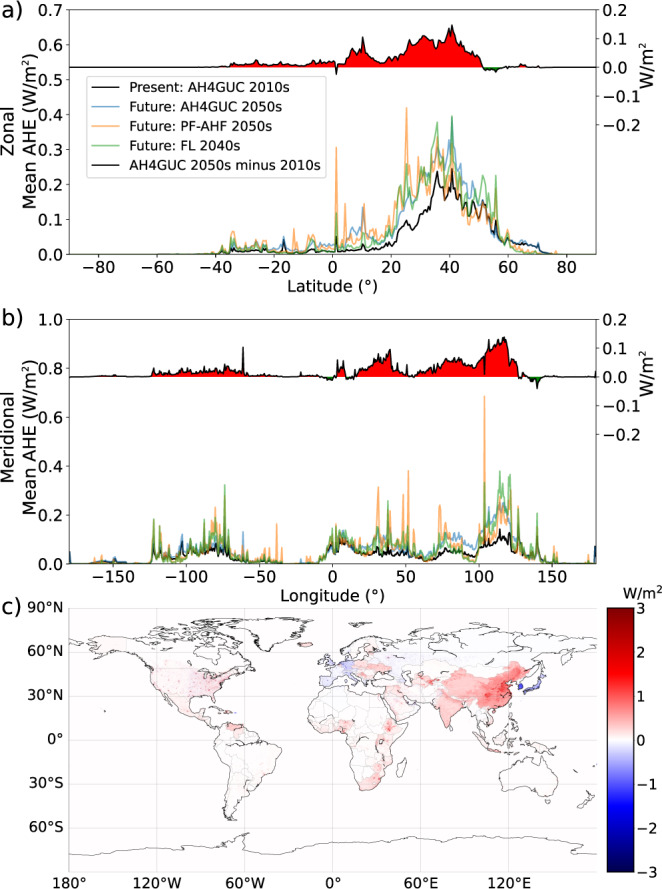
Zonal mean (**a**) and meridional mean (**b**) of present and future anthropogenic heat emission (left-hand side vertical axis); and the zonal and meridional mean difference (upper chart in a and b) between the present 2010s and future 2050 s calculated from AH4GUC (right-hand side vertical axis). Red-filled and green-filled regions correspond to a zonal or meridional 2050 s increase and decrease of emissions, respectively. All datasets resampled to FL resolution (0.5°) and statistics estimated at 0.5° intervals. (**c**) shows a global map of the difference between 2050 s and 2010s of the AH4GUC in native resolution.

Figure 6 shows the differences in future estimates of AHE from AH4GUC, PF-AHF, and FL. Note that in FL, the 2040 s estimate is shown instead due to availability. On the primary vertical axis (left-hand vertical axis), the same analyses as Fig. 3 is shown showing zonal and meridional mean of future AHE from the “top-down” datasets (for reference with the present, the 2010s AHE from AH4GUC is also shown). On the secondary vertical axis (right-hand vertical axis), the difference of AHE between the 2050 s and 2010s according to AH4GUC is shown. Comparing the future datasets, the global averages are 0.047 W/m^2^, 0.049 W/m^2^, and 0.050 W/m^2^ for FL, PF-AHF, and AH4GUC, respectively. The excess 0.001 W/m^2^ in AH4GUC compared with the other datasets comes from larger estimates at detected hot spots located at the middle and higher latitudes of the northern hemisphere as explained earlier and in Fig. 3. Moreover, inputs for the future projection of AHE from AH4GUC were based on worst-case climate change scenarios (RCP8.5 and SSP3, see *Methods*) with additional consideration for urban sprawling. To supplement existing future scenarios provided by PF-AHF and FL, AH4GUC provides an extreme AHE scenario that considers the detailed representation of urbanization changes to the future. From the methodology presented in this work, other climate-change scenarios may also be considered.

The projected increase in AHE from 2010s to 2050 s reveals two distinct increases along the latitude (Fig. 6a), namely: from the equator up to 15° North, and at the mid-latitudes. Along the longitude (Fig. 6b), the increase is also more significant east of 0° longitude. A map of the changes in AHE from the 2010s to 2050 s is shown in Fig. 6c with the color range of visualized AHE reduced for visibility. Large projected increases in AHE are located in Asia, Africa, and some countries in South America and North America. In the United States, the West, Northwest, and Southwest states will have increasing emissions while decreases are felt for some less urbanized areas in the Mid-West, South East, Mid-Atlantic, and North East US. In Europe, the western countries will generally be emitting lower AHE in the 2050 s compared with the 2010s; while the AHE of Eastern Europe is expected to increase. A decrease in AHE by the 2050 s is most prominent in South Korea and Japan. Decreases in AHE are attributed mainly to population decline throughout the country and depopulation in rural areas.

Another significant difference between existing “top-down” approaches and AH4GUC is the consideration of point sources using VIIRS Nightfire datasets (see *Methods*). In the AH4GUC, a portion of the annual energy loss was allocated to the grids representing point sources of each country. 9.17% of all 29,910 powerplants (estimated using zonal statistics of the surrounding powerplants with a radius of 10-km) listed in the Global Powerplant Database^23^ were found to coincide with the detected point sources suggesting that not all powerplants have detectable heat emissions from VIIRS. An example of a powerplant in the Netherlands coinciding with the detected point source is shown in Supplementary Figure S6. On the other hand, many powerplants were also found to have detected point sources surrounding it by more than 10-km away (Supplementary Figure S7). This suggests the possibility that the geospatial location of powerplants listed in the Global Powerplant Database^23^ may also refer to headquarters of powerplants slightly located farther from its high-heat-emitting components.

## Usage Notes

We hope that this AH4GUC, which is a major improvement from previously estimated “top-down” datasets and that of Dong *et al*.^10^, will provide researchers and urban planners with detailed distribution of anthropogenic heat emissions of the present and the 2050 s period. Its future AHE projection, which considers urban growth and population projections under worst-case scenario, will be useful in climate change modeling where urbanization is not well represented.

With the advancement of computational speed, general circulation models (GCM), which are used to model the effects of climate change, are becoming more capable of wide-scale simulations at high-spatial resolutions. Alongside this, high-spatial maps of urban parameters, which can serve as surface boundaries to GCMs urban surface models, are becoming necessary. Global maps of future AHE, such as the AH4GUC, can contribute to existing future scenarios (e.g. PF-AHF and FL).

This dataset is highly recommended to users who wish to estimate the possible changes in the global distribution of AHE between the present and the 2050 s. When investigating for specific cities, users have to carefully inspect both AH4GUC and DONG datasets and decide suitability for the city. Furthermore, given that the worst-case defines the future AHE scenario in this dataset, adaptation and mitigation strategies were not assumed. The primary reason for this was that urban sprawling data (used as input) was mainly generated from historical urban cover information and an SSP3 pathway. Furthermore, users have to be aware that this was derived using a “top-down” approach and is expected to be less accurate from those that were estimated from “bottom-up” approaches (see *Technical Validation*). Hence, adjustments may be needed for certain regions. The AH4GUC will serve as an alternative data or additional option for locations with inaccurate or missing AHE records.

Finally, AH4GUC is the first “top-down” dataset to incorporate global point sources automatically derived from VIIRS Nightfire^32^ outputs. According to Elvidge *et al*.^32^, the VIIRS Nightfire captures high-radiant emissions associated with gas flares, biomass burning, volcanoes, and industrial sites such as steel mills. Although we processed the long-term VIIRS Nightfire dataset to detect more frequently occurring emissions such as those from industrial sites, the algorithm introduced in this work may be further improved in the future.

A strong limitation can be found in the usage of urban growing scenario to map future population. To estimate future population distribution, urban an growing probability map was needed (see “Methods”). During the construction of AH4GUC, GUGPS was the only latest publicly available dataset that provides a 1-km urban growing probability projection based on the historical growth of cities. We assumed that combining this dataset with the total country-level population assumed from the SSP will force the future AHE intensities to be somehow consistent with the designed future climate scenario (e.g. SSP3-RCP8.5). This assumption remains uncertain since urban growth itself should also be linked with the defined SSP.

The above limitations may be addressed by improving the SLEUTH model used in GUGPS or by incorporating more recent urbanization scenarios or datasets that are consistent with SSP. GUGPS was constructed using the SLEUTH model, which utilizes growth coefficients derived from historical geospatial datasets (see “Methods” section). The growth coefficients^33^ physically represent various mechanisms of urbanization (e.g. diffusion, growth along roads). The coefficients may be manually adjusted to match the definition of the SSP. To achieve this, a priori understanding of the underlying assumptions of the SSP and the individual effects of each growth coefficient to urban growing must be known. Specific future policies of the country may also be introduced by adjusting the said growth coefficients in SLEUTH. Another way to be overcome the said limitation would be to utilize more recent urbanization datasets that are more compatible with SSPs. The work by Chen *et al*.^34^, which provides different conditions of global urban cover changes for each SSP, may be used to replace the GUGPS dataset or combine with it to provide urbanization probability maps.

## Supplementary information

Supplementary Information

## Data Availability

The codes for constructing the dataset were written in Python language and can be downloaded from https://urbanclimate.tse.ens.titech.ac.jp/ or may be requested directly from the corresponding author. The inputs used to construct the present and future AHE datasets are all publicly available online with sources cited within this manuscript. Specific pre-processed inputs may be requested from the corresponding author upon request.
