# Supplementary material for: Global 1-km present and future hourly anthropogenic heat flux
Source: Sci Data. 2021 Feb 22;8:64. doi: 10.1038/s41597-021-00850-w (PMC7900113; doi:10.1038/s41597-021-00850-w)
Supplement: Supplementary file 1 — Supplementary Information [file 41597_2021_850_MOESM1_ESM.docx]

### Title

Global 1-km present and future hourly anthropogenic heat flux

### Authors

Alvin Christopher Galang Varquez^1^, Shota Kiyomoto^1^, Do Ngoc Khanh^1^, Manabu Kanda^1^

**Affiliations**

1. Department of Transdisciplinary Science and Engineering, Tokyo Institute of Technology

corresponding author(s): Alvin Christopher Galang Varquez (varquez.a.aa@m.titech.ac.jp)

This document contains supplementary information to the main article.

**Table of Figures**

[Figure S1 Same as Fig. 3 of the manuscript without DONG dataset and all datasets resampled (by averaging) to the same resolution as FL (2.5’ or 2.5 arc-minutes) 2](#_Toc58920136)

[Figure S2 Annual-average AHE (W/m^2^) representation at commercial area of Jakarta from a) DONG and b) AH4GUC. 3](#_Toc58920137)

[Figure S3 Annual-average AHE (W/ m^2^) representation over a region in Russia. The “top-down” datasets are a) AH4GUC, b) PF-AHF, c) AH-DMSP, and d) FL. 3](#_Toc58920138)

[Figure S4 Annual-average AHE (W/ m^2^) representation of the region bounded by a green box in Fig. S3. The “top-down” datasets are a) AH4GUC, b) PF-AHF, c) AH-DMSP, and d) FL. 4](#_Toc58920139)

[Figure S5 Annual-average AHE (W/ m2) representation of the Novokuznetsk City, Russia bounded by a green box in Fig. S4. The “top-down” datasets are a) AH4GUC, b) PF-AHF, c) AH-DMSP, and d) FL. A corresponding satellite image was downloaded from the World Imagery of ESRI (http://server.arcgisonline.com/arcgis/rest/services/World_Imagery/MapServer) 5](#_Toc58920140)

[Figure S6 Case when the detected point source area (region filled with transparent red) coincides with a designated power plant (e.g. Ijmond, a powerplant in the Netherlands, http://powerplants.vattenfall.com/ijmond, accessed June 2020). Underlying basemap was taken from Google satellite static image. 6](#_Toc58920141)

[Figure S7 Case when the detected point source area (region filled with transparent red) lies a few kilometres away from a designated powerplant (e.g. Guohua Wulate Zhongqi Chuanjing Phase 2, a powerplant in China, https://cdm.unfccc.int/Projects/redirector?ref=4303, accessed June 2020). Underlying basemap was taken from Google satellite static image. 6](#_Toc58920142)


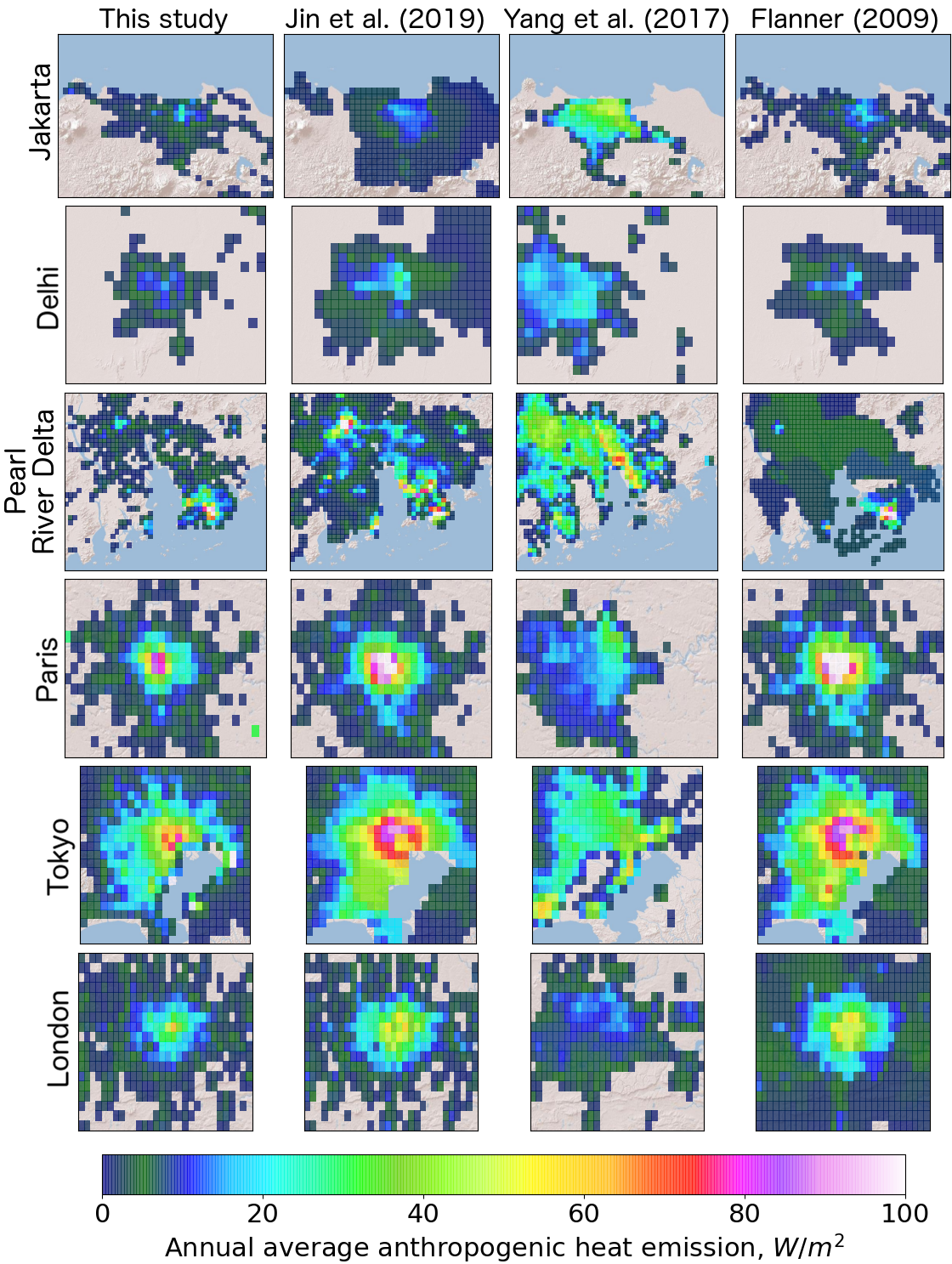


Figure S1 Same as Fig. 3 of the manuscript without DONG dataset and all datasets resampled (by averaging) to the same resolution as FL (2.5’ or 2.5 arc-minutes)


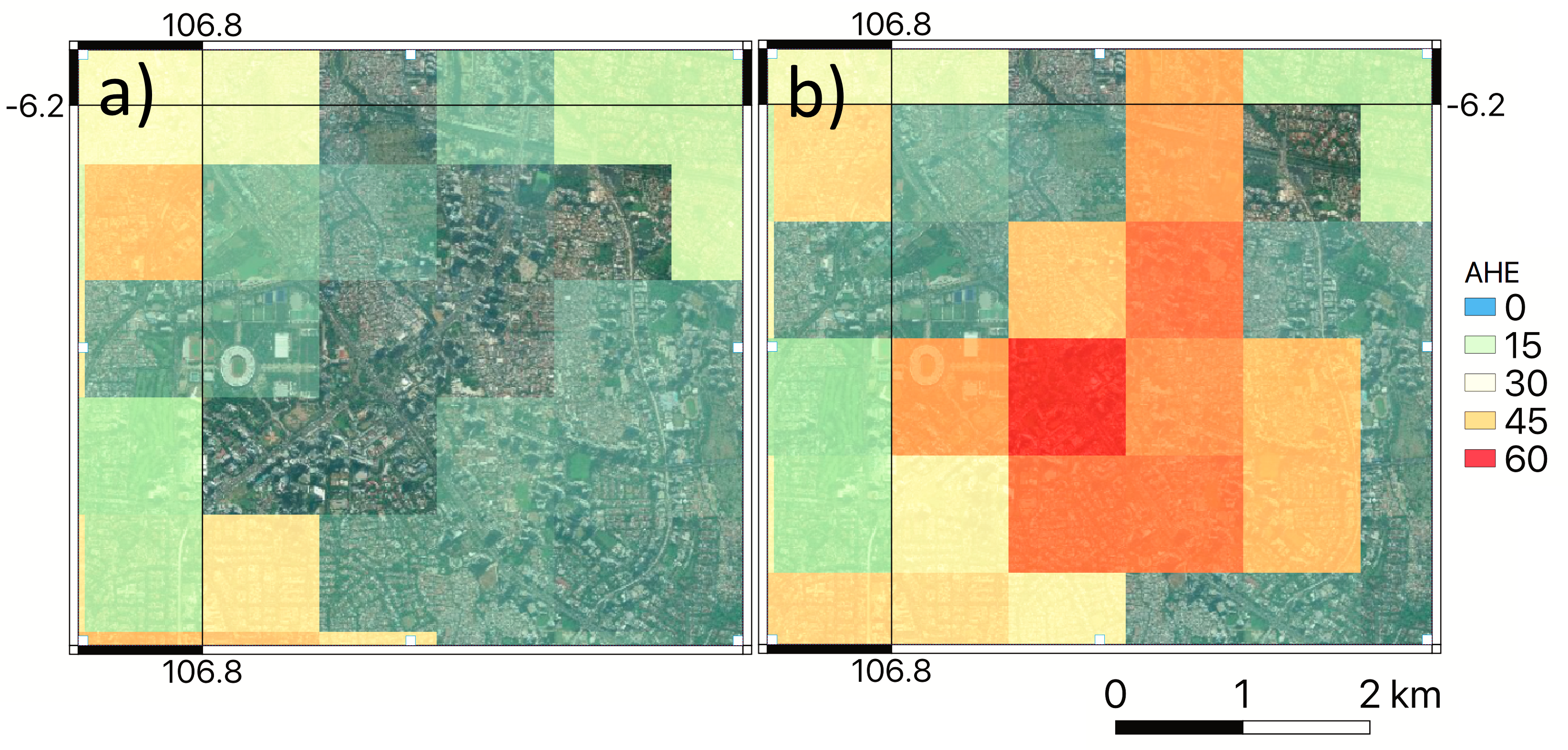


Figure S2 Annual-average AHE (W/m^2^) representation at commercial area of Jakarta from a) DONG and b) AH4GUC.


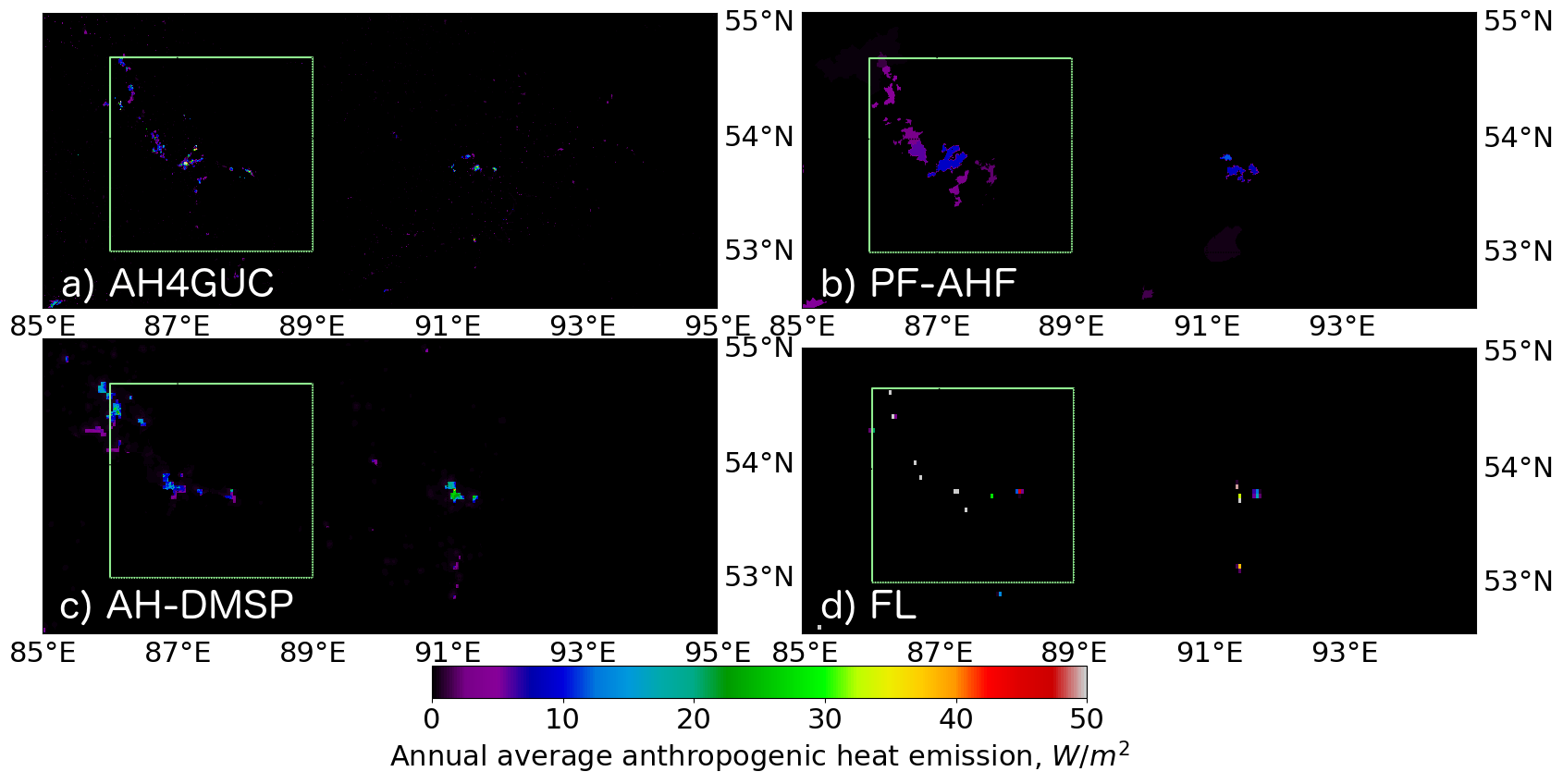


Figure S3 Annual-average AHE (W/ m^2^) representation over a region in Russia. The “top-down” datasets are a) AH4GUC, b) PF-AHF, c) AH-DMSP, and d) FL.


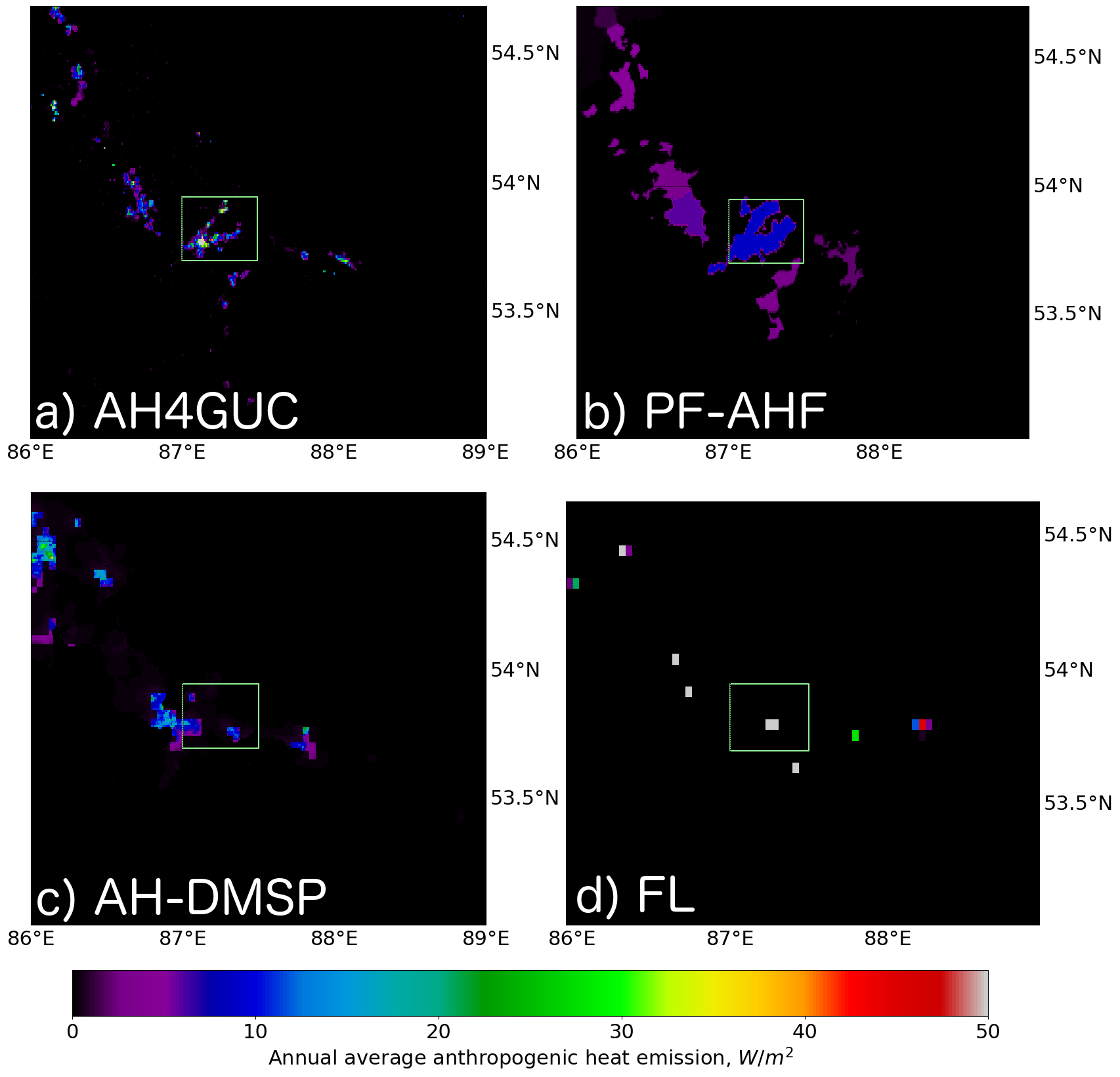


Figure S4 Annual-average AHE (W/ m^2^) representation of the region bounded by a green box in Fig. S3. The “top-down” datasets are a) AH4GUC, b) PF-AHF, c) AH-DMSP, and d) FL.


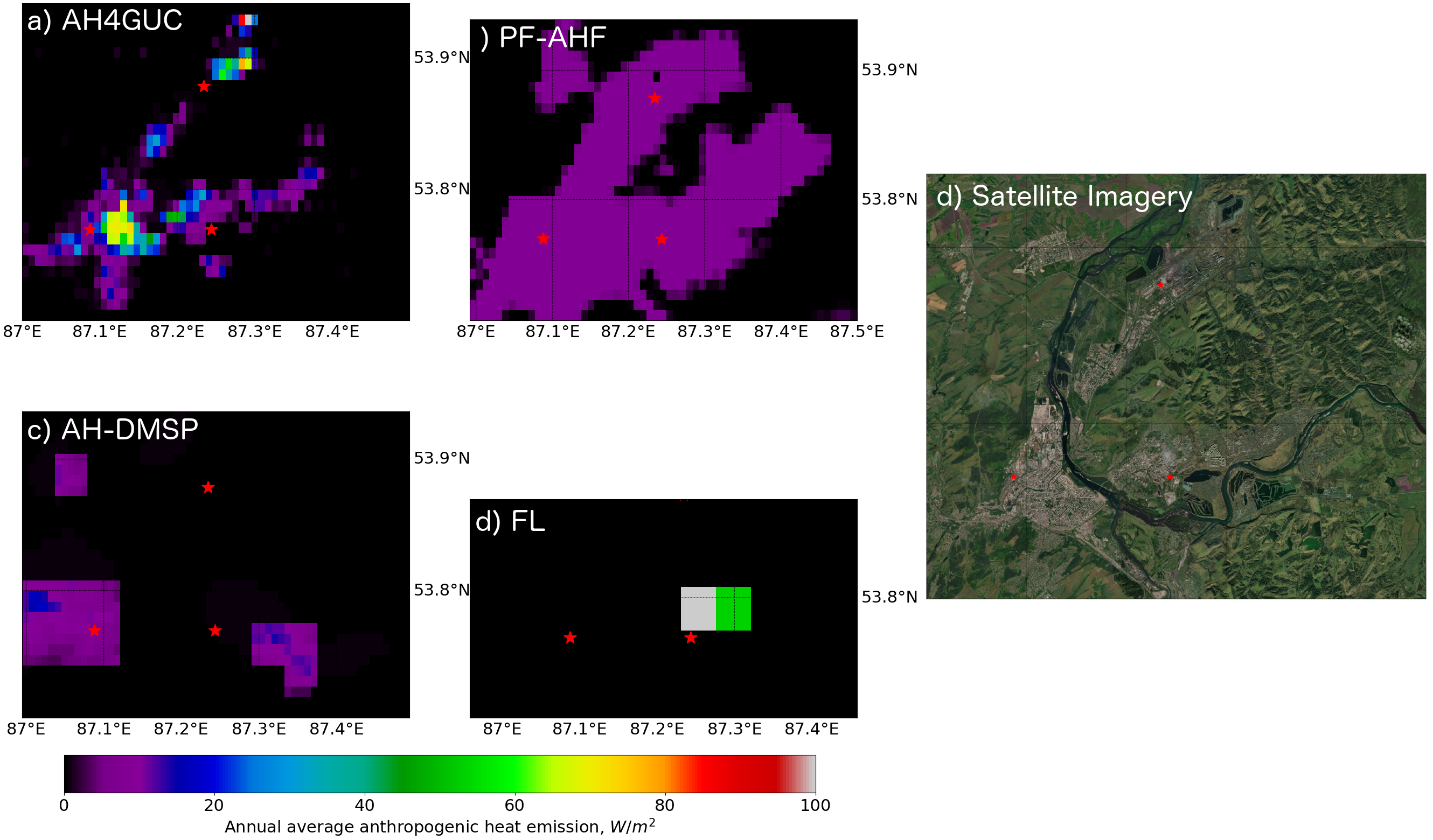


Figure S5 Annual-average AHE (W/ m2) representation of the Novokuznetsk City, Russia bounded by a green box in Fig. S4. The “top-down” datasets are a) AH4GUC, b) PF-AHF, c) AH-DMSP, and d) FL. A corresponding satellite image was downloaded from the World Imagery of ESRI (<http://server.arcgisonline.com/arcgis/rest/services/World_Imagery/MapServer>)


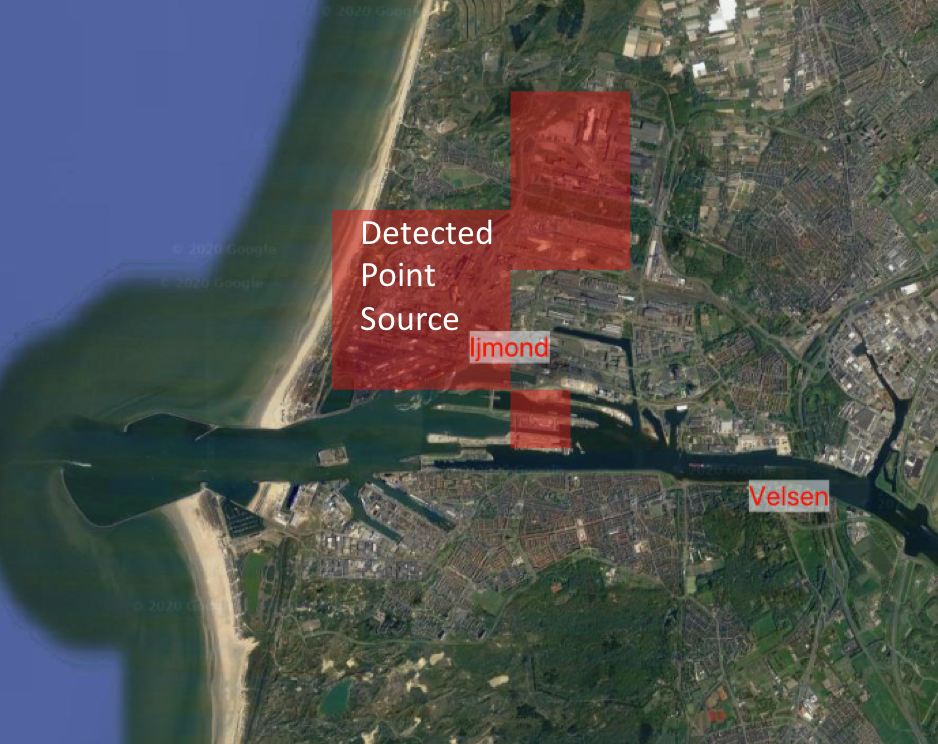


Figure S6 Case when the detected point source area (region filled with transparent red) coincides with a designated power plant (e.g. Ijmond, a powerplant in the Netherlands, <http://powerplants.vattenfall.com/ijmond>, accessed June 2020). Underlying basemap was taken from Google satellite static image.


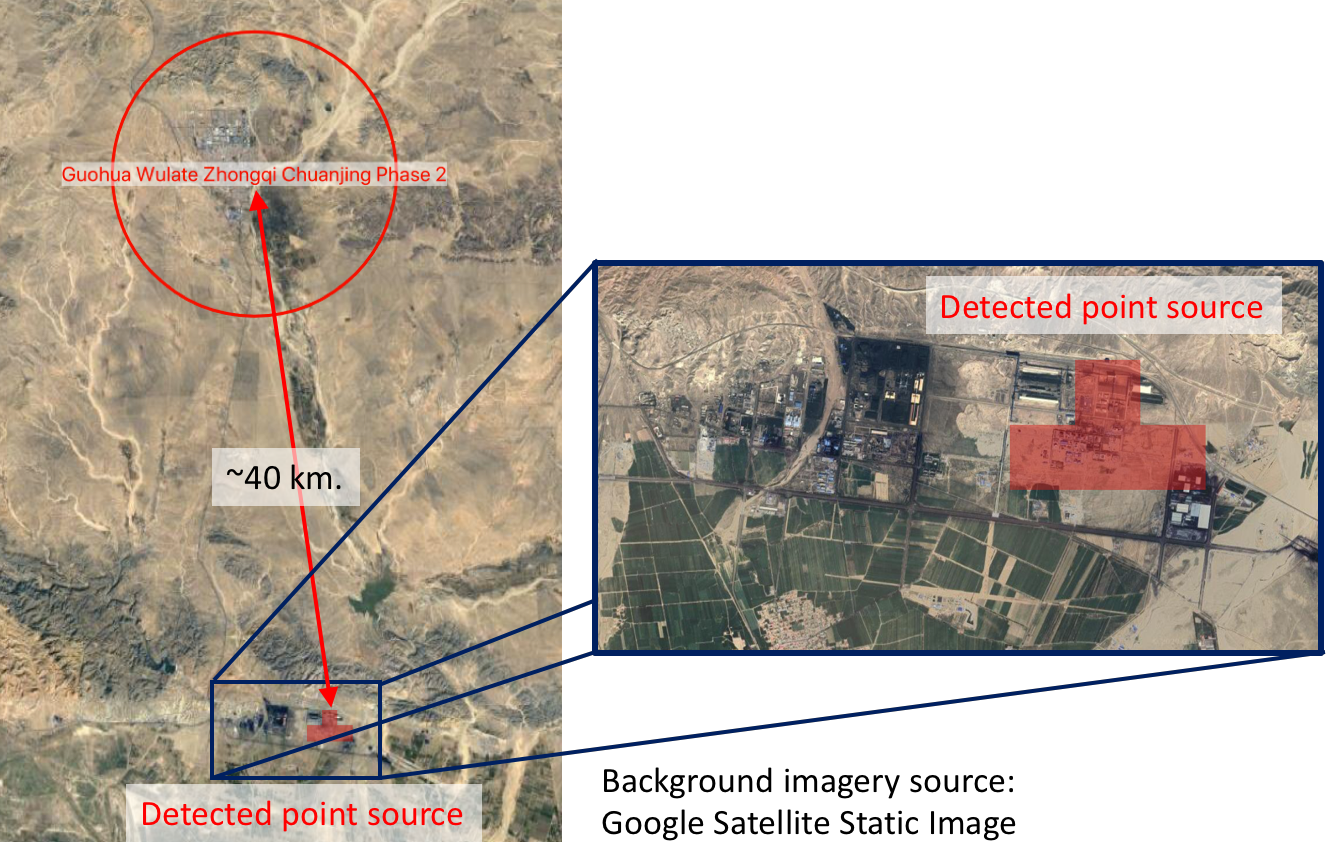


Figure S7 Case when the detected point source area (region filled with transparent red) lies a few kilometres away from a designated powerplant (e.g. Guohua Wulate Zhongqi Chuanjing Phase 2, a powerplant in China, <https://cdm.unfccc.int/Projects/redirector?ref=4303>, accessed June 2020). Underlying basemap was taken from Google satellite static image.

Table S1 List of RCP8.5 models used to generate the monthly surface air temperatures

| ACCESS1-0 | ACCESS1-3 |
| --- | --- |
| BNU-ESM | CCSM4 |
| CESM1-BGC | CESM1-CAM5 |
| CESM1-WACCM | CMCC-CESM |
| CMCC-CM | CMCC-CMS |
| CNRM-CM5 | CSIRO-Mk3-6-0 |
| CanESM2 | EC-EARTH |
| FGOALS-g2 | FIO-ESM |
| GFDL-CM3 | GFDL-ESM2G |
| GFDL-ESM2M | GISS-E2-H |
| GISS-E2-R | HadGEM2-AO |
| HadGEM2-CC | HadGEM2-ES |
| IPSL-CM5A-LR | IPSL-CM5A-MR |
| IPSL-CM5B-LR | MIROC-ESM |
| MIROC-ESM-CHEM | MIROC5 |
| MPI-ESM-LR | MPI-ESM-MR |
| MRI-CGCM3 | NorESM1-M |
| NorESM1-ME | bcc-csm1-1 |
| bcc-csm1-1-m | inmcm4 |
